# Influence of Torsion on Matteucci Effect Signal Parameters in Co-Based Bistable Amorphous Wire

**DOI:** 10.3390/ma12030532

**Published:** 2019-02-11

**Authors:** Tomasz Charubin, Michał Nowicki, Roman Szewczyk

**Affiliations:** Warsaw University of Technology, Institute of Metrology and Biomedical Engineering, ul. Sw. A. Boboli 8, 02-525 Warsaw, Poland; m.nowicki@mchtr.pw.edu.pl (M.N.); r.szewczyk@mchtr.pw.edu.pl (R.S.)

**Keywords:** Matteucci effect, magnetoelastic effect, amorphous wire, magnetic bistable wire, Barkhausen Jump

## Abstract

The Matteucci effect (ME) is one of the lesser-known magnetomechanical effects and is most prominent in bistable amorphous wires. It has some experimental applications—Matteucci effect-based magnetic field sensors are very easy to produce and have inherently linear, hybrid analog/digital output signal. The effect is still poorly understood, however, and although it relies on torsion of the wire to manifest, there is no available model, or much experimental data, which would quantitatively connect the ME with the sample twist. In this paper, experimental characteristics of ME signal parameters dependence on torsion in Co-based amorphous bistable wire are presented. The results hint at possible applications, such as rotation or critical current sensors, as well as the necessity of torsion control in the development of ME magnetic field sensors.

## 1. Introduction

Matteucci effect (ME) is one of many magnetomechanical effects described by early investigators of electromagnetic phenomena. In ME, the circumferential magnetization component of a given material, mostly in the form of wire or tube, switches with the application of an axial magnetic field. This change induces a pulse of electrical voltage across the sample for each change of magnetization state. These pulses are called the Matteucci voltage [1]. It was described by Carlo Matteucci in 1858 [2] and was half forgotten until it was observed again in twisted amorphous ferromagnetic wires and ribbons [3,4]. The ME was later utilized in the development of various devices, such as magnetic field sensors [5], rotational speed sensors [3], critical current sensors [6], fast pulse generators [3] and in high precision magnetic quartz sensors, which, using a new switching sensing principle, have high sensitivity and temperature compensation [7]. The knowledge about the effect is not widespread, however, which significantly limits its utilization. There are also few works with detailed measurement results or model proposals, which calls for additional experimental investigations. Based on the results, computer simulations of the effect could be performed, which will be essential for design and optimization of novel ME devices [8]. The Wiegand effect [9] is similar from an application point of view but relies on axial magnetization change and requires additional pickup coils. 

The ME depends on circumferential magnetization M_Φ_ change under the influence of axial magnetic field H_z_. This in turn occurs only when the sample exhibits helical magnetization. Helical magnetization vector is a superposition of axial and circular vectors and is procured either by applying torsion to the sample or by annealing a twisted sample beforehand. 

For Co-based, magnetically bistable amorphous wires, the ME pulses are sharp and short. If there is only one Large Barkhausen Jump, the magnetic domains inside the sample rotate together after exceeding the coercive (triggering) field value [1,3]. The material used in this investigation is declared by the manufacturer to be of a large Barkhausen jump type [10]. It is, however, known that often Co-based wires show bistability because the domains are stabilized through the perminvar effect, and the induced signal only looks similar to a large Barkhausen jump [11]. 

In previous papers, our team developed and tested novel pulse-shifted Matteucci Fluxgate [12,13] working in the time-delay mode. Primary harmonics of the sensor signal were also investigated, due to a widespread second harmonic sensing technique for signal extraction from magnetic fluxgate sensors [14].

However, there is an open question about the influence of torsion on the ME in Co-based amorphous wires. It was known that the torsion induces helical magnetization of the sample and allows for the effect to manifest, but there is a lack of information about the character of this influence. This paper is filling this gap with detailed experimental characteristics of ME voltage peaks parameters vs. the torsion of the sample. The exact mechanism of these non-linear magnetoelastically coupled relationships will require further works; however, the presented results allow for setting the framework for future modeling of the effect, as well as potential sensor applications.

## 2. Materials and Methods

### 2.1. Sample

**Figure 1:**
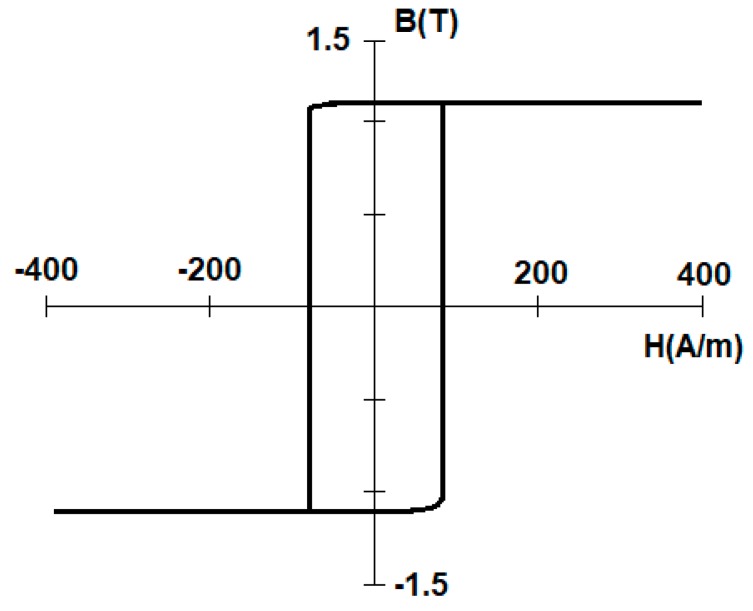
Rectangular hysteresis loop of used amorphous wire [10].

The sample was an amorphous Co-Fe-based wire manufactured by Aichi Steel Corporation (manufacturer symbol 101DC5T, Higashiura, Japan), 101 µm in diameter, length 60 mm. The alloy was chosen due to its rectangular hysteresis loop, as the prediction was that bistability would provide the best ME signal, due to the almost instantaneous magnetization direction change (large Barkhausen jump). A sample hysteresis loop from the manufacturer’s datasheet is presented in Figure 1 [10].

The saturation magnetostriction was not determined due to the lack of exact composition of the used wire; normally Co-Fe-Si-B alloys exhibit saturation magnetostriction of approx. −0.5 to 0 ppm in the as-cast state [15].

**Figure 2:**
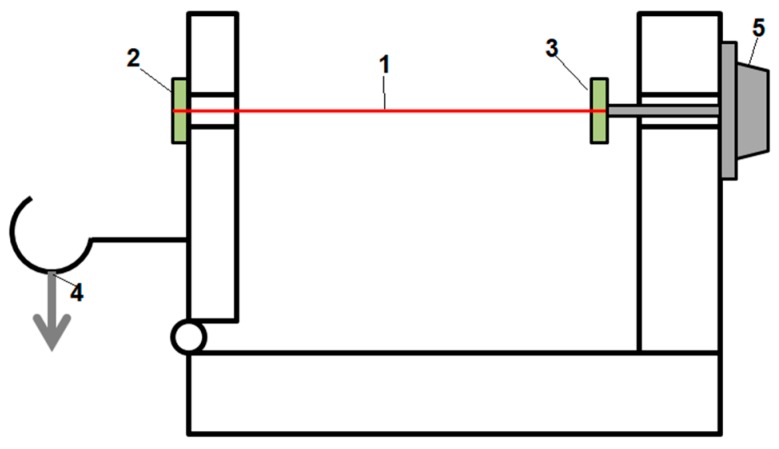
Schematic of the mounting of the wire sample. 1—amorphous wire, 2—fixed electrode, 3—rotating arm with electrode, 4—applied load, 5—precise rotation knob.

The amorphous wire was soldered to electrodes using Lichtenberg alloy, which melts at 90 °C, to avoid any crystallization caused by heat, then glued to external rotating arms, which would apply torsion and axial force to the wire. A detailed schematic is presented in Figure 2. The sample was exposed to rotation of −2.5 to +2.5 turns, which equals to shear strain of −0.013 to 0.013, and the resulting applied axial stress equaled 70 MPa.

### 2.2. Measurement System

The measurement system consisted of a PC equipped with a custom-made program developed in National Instruments (Austin, TX, USA.) LabVIEW software and a National Instruments PCI-6221 data acquisition (DAQ) card, to which the voltage signals from current measurement (1 Ω resistor in series with magnetizing current) and the sample were connected. The DAQ card also provided voltage amplification, so as not to introduce additional delays and noise provided by external amplifiers. Every measurement was taken 20 times and averaged into one result and its standard deviation.

**Figure 3:**
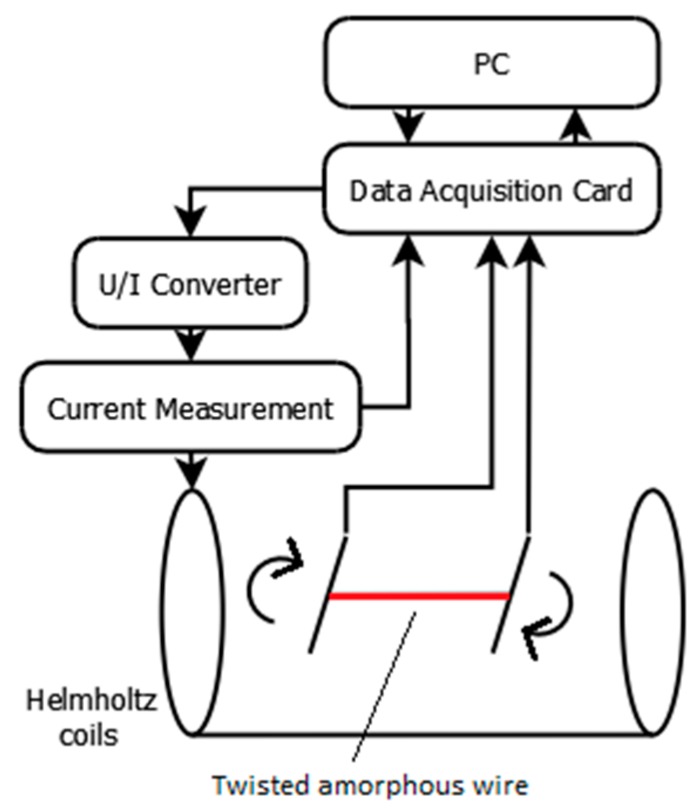
Simplified schematic block diagram of the measurement system.

The sample with its mounting was put inside a Helmholtz coil pair to ensure a homogeneous magnetizing field. The coils were powered from a KEPCO (New York, NY, USA) BOP 36-6M U/I converter controlled by the DAQ card and were magnetized using a triangle wave with amplitude of 200 A/m and frequency of 5 Hz. The ME voltage was then measured by the DAQ, then recorded and displayed on the PC. The measurement system block schematic is presented in Figure 3.

## 3. Measurement Results

### 3.1. Voltage Pulses from Matteucci Effect

In the article the following parameters of ME pulses in the function of torsion were measured:

- Triggering field value (A/m)—the magnetizing field value, at which the large Barkhausen jump occurs
- Voltage amplitude value (mV)—the maximum voltage value of the pulse
- Pulse width (μs)—defined as the pulse width at half the maximum voltage value

**Figure 4:**
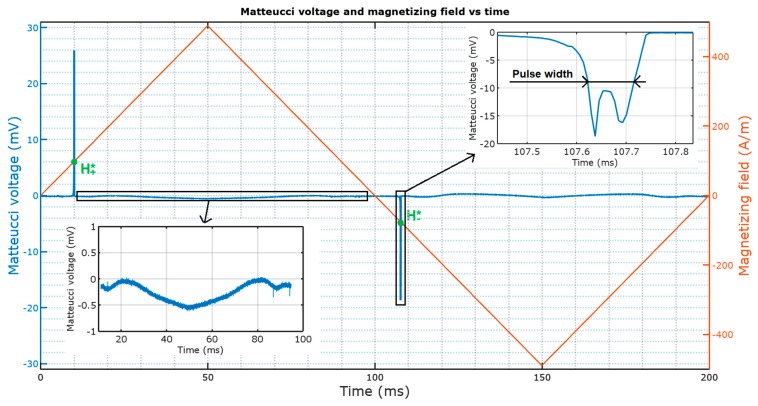
Measured values on exemplary Matteucci effect (ME) pulse plot ($H_{+}^{*}$—positive peak triggering field, $H_{-}^{*}$—negative peak triggering field).

A diagram explaining these values on an exemplary plot of Matteucci voltage and magnetizing field vs. time is presented in Figure 4.

### 3.2. Triggering Field Dependence

**Figure 5:**
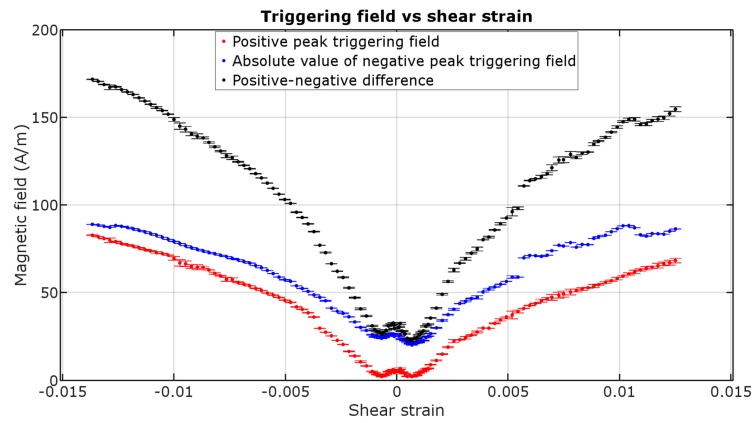
Triggering field of positive peak, negative peak and their difference caused by shear strain (±1 std. deviation).

Figure 5 presents the triggering field in function of shear strain of the sample. The dissimilarity between the negative and positive peak values are caused by the non-zero earth’s magnetic field (~10 to 30 A/m in one axis), which was not controlled for in this experiment.

The values of field in which the sample changes its magnetization tend to rise with increase of torsion. It is expected due to the increase in coercive field of magnetic materials with the increase of torsion, as was reported in previous work [16]. The characteristic is near second degree polynomial, with some outliers. Near zero shear strain the results are more chaotic, due to extremely small ME amplitude and higher pulse width. However, the small double-peak section in ±0.001 shear strain range was repeatable. The asymmetry between directions of rotation may be caused by residual stresses from manufacture, because the samples were not annealed before the measurements.

### 3.3. Peaks’ Amplitude Dependence

**Figure 6:**
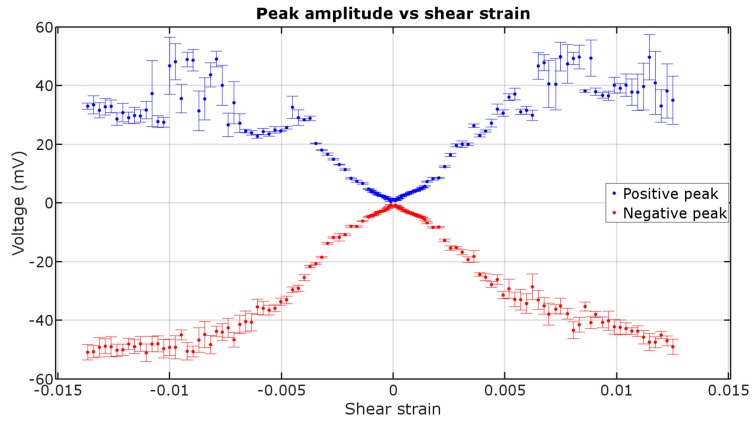
Matteucci voltage amplitude in function of shear strain (±1 std. deviation).

Figure 6 presents the amplitude of ME voltage pulses in function of shear strain. The amplitude increases to a certain value of shear strain, then it stabilizes.

Amplitude of the voltage peaks is a quantitative parameter that could be used in sensor application, but the physical meaning of the voltage generated is a change in circumferential magnetization M_Φ_, which is described by the following equation [17]:

(1) $$V\left(t\right)=0.35\;al\mu_{0}\left(\frac{\partial M_{\emptyset }}{\partial t}\right),$$

where, *V*(*t*)—generated voltage, *a*—radius of the wire, *l*—length of the wire, *μ*_0_—magnetic permeability constant, and *t*—time. Transforming Equation (1), the change in circumferential magnetization can then be described by the following equation:

(2) $$\langle M_{\emptyset }\rangle =\frac{\int Vdt}{0.35\;al\mu_{0}},$$

**Figure 7:**
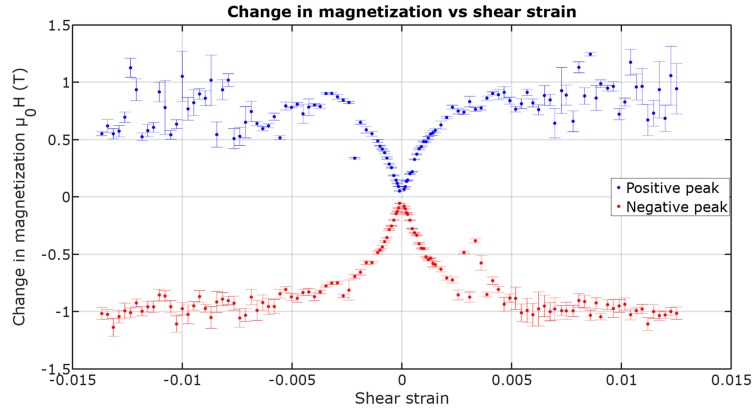
Magnetization change in function of shear strain (±1 std. deviation).

Following Equation (2), by integrating only the ME voltage peaks, a plot describing change in magnetization caused by shear strain during each magnetization change was presented in Figure 7. Note that the integrated signal for each measurement was taken from a single plot, not from a plot averaged from 20 cycles.

The change of magnetization saturates near the value of ~1.1 T for negative peaks, which would match the saturation flux density given by the manufacturer.

### 3.4. Peaks’ Width Dependence

**Figure 8:**
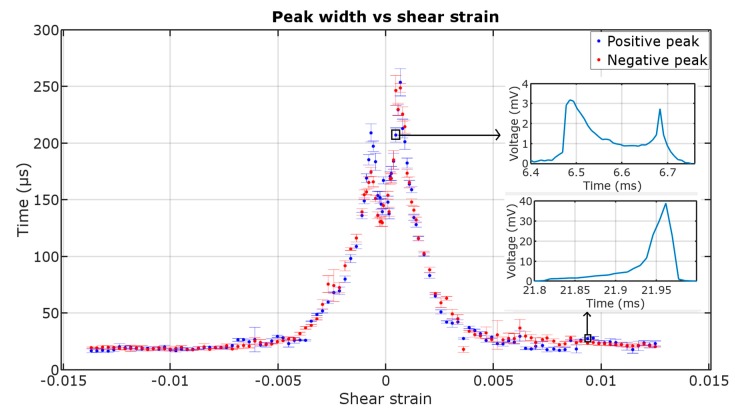
Dependence of the ME peaks’ width on shear strain (±1 std. deviation, insets show the voltage signal vs. time plot for two exemplary points—low shear strain and high shear strain region).

Figure 8 presents the width of ME voltage pulses in function of shear strain. The pulse width, after initial growth, starts to decrease and stabilizes at a certain value for both positive and negative peaks. 

## 4. Discussion

The magnetic ME voltage response is stronger and faster when increasing the applied shear strain, but also those magnetization jumps require higher triggering field value to occur. The increase of coercive field occurs due to the stress between magnetic domains in micro-scale, which would require higher force to turn themselves parallel to the magnetizing field.

The higher the shear strain, the higher the peaks’ voltage. For lower values of shear strain, the effect is linear until reaching saturation. The saturation of the peak amplitude is due to achieving saturation induction of the circular magnetization in the sample, which is near the 1.1 T declared by the manufacturer for longitudinal magnetization [10]. This means that the domain rotation is nearly 90 degrees during each magnetization jump. This means that nearly 0.005 shear strain has to be applied for the sample’s domains to form an easy magnetization direction close to +45 degrees between axial and circumferential position, and then rotate to nearly −45 degrees after each jump. 

The peak width is stable in the high shear strain region. In the low shear strain region (<0.002) the peaks appear as though they were dual, one directly after another, which increases the peak width. By increasing the shear strain, the two peaks come closer to each other and join into one peak with a higher amplitude, stabilizing at ~20 μs width. This behavior may be explained by non-simultaneous domain wall propagation starting from both ends independently and hints at more than one magnetization jump in the sample. The peak width may also change with the shear strain due to the strength of the local anisotropy fields’ change, which in turn will affect the speed of domain wall propagation and therefore the dynamics of the switching event.

## 5. Conclusions

The research indicated that torsion has a significant influence on the properties of a sensor based on amorphous wire utilizing ME voltage signal. The presented experimental characteristics of ME voltage parameters in function of shear strain applied to sample show phenomena, such as the peak width and amplitude saturation. This hints at complex magnetoelastic behavior of the materials exhibiting ME, which requires further research. 

Some of the presented results allow for development of novel ME-based sensors, whereas the variability of ME parameters with rotation must be taken into account while developing novel ME fluxgates for magnetic field sensing.

The triggering field value rises with an increase of torsion. This phenomenon could, for example, be used to make a rotation sensor, given the significant and monotonic increase of H^*^. It is also important for other applications, as, due to the magnetoelastic effect of torsion, the triggering field of this amorphous wire can be tuned for specific value. It would therefore allow for construction of critical current sensor with adjustable triggering current.

The phenomenon could be also used to measure the coercivity of an amorphous wire during its production. The obtained triggering field values are raised with shear strain, and for 0° rotation there is no ME. The values obtained for small shear strain are close to the no-rotation coercivity but are not exact. On the other hand, it could be possible to measure the triggering fields for two values of rotation and extrapolate it downwards given its near-linear relationship.

The amplitude of signal rises monotonically with shear strain and stabilizes after the value of ~0.005. This phenomenon could be used to make a small-angle rotation sensor with distinguishable angle-direction, after ~0.001 shear strain prestressing.

The shape of the peak (double maximum with variable width) can itself be used for assessment of magnetization behavior of amorphous wire samples. The peak width changes in non-uniform magnetic fields and could be used in magnetovision scanners. Future research shall be given concerning the dual peaks (as seen in the insets of Figure 4 and Figure 8), as the shape of the peaks changes with the increase of torsion and with the application of a non-uniform magnetic field.

Sensors based on ME are working on the principle of the magnetic field momentary exceeding a certain value, not of continuous analog signal generation, like magnetoresistive sensors. Without using any external power, they can detect AC fields. Also, transducers based on ME have a perfect linear characteristic with the lower field measurement range limited by the wire’s coercive field and the upper field measurement range limited by the magnetizing coil (which in theory is not limited). The disadvantages include low stability of the peaks’ amplitude in a high shear strain region (>0.005), compared to a low shear strain range.
